# Does the Use of Viscoelastic Hemostatic Assays for Periprocedural Hemostasis Management in Liver Disease Improve Clinical Outcomes?

**DOI:** 10.1016/j.tmrv.2024.150823

**Published:** 2024-07

**Authors:** Suzanne Maynard, Elizabeth Marrinan, Lara Roberts, Simon Stanworth

**Affiliations:** a NIHR Blood and Transplant Research Unit in Data Driven Transfusion Practice, Nuffield Division of Clinical Laboratory Sciences, Radcliffe Department of Medicine, University of Oxford, Oxford, UK; b Institute of Pharmaceutical Sciences, King's College London, UK; c Department of Haematological Medicine, King's College Hospital NHS Foundation Trust, London, UK; d Department of Haematology and Transfusion Medicine, NHSBT and Oxford University Hospitals NHS Foundation Trust, Oxford, UK

**Keywords:** Cirrhosis, Hemostasis, Liver, Thromboelastography, Thromboelastometry

## Abstract

Routine hemostasis parameters such as prothrombin time and fibrinogen are frequently abnormal in patients with chronic liver disease and have been demonstrated to be poor predictors for periprocedural bleeding. Alterations in procoagulant and anticoagulant factors in this population result in a state of rebalanced hemostasis, which is not reflected by routine hemostatic measures. Viscoelastic hemostatic assays (VHA) present a point of care measure of global hemostasis with an emerging role in guiding transfusion in the liver transplant setting. The potential role for VHA in guiding periprocedural transfusion is unknown. Here we critically appraise the available limited evidence on the use of VHA to guide prophylactic treatment in patients with cirrhosis undergoing procedures. We assess whether the impact of a VHA-guided approach improves clinical outcomes. Suggested areas for future research with a focus on clinically relevant outcomes, particularly periprocedural bleeding, are highlighted.

## Highlights

• Changes in pro/anticoagulant factors in cirrhosis result in rebalanced hemostasis.
• Routine hemostatic measures do not predict periprocedural bleeding in cirrhosis.
• Point of care viscoelastic hemostatic assays (VHA) assess global hemostasis.
• Evidence to support VHA use in predicting procedural bleeding in cirrhosis is limited.
• Whilst VHA-use might reduce blood component use, this may not impact on bleeding.

## Introduction

Abnormalities of routine measures of hemostasis such as prolonged prothrombin time (PT) / international normalized ratio (INR) or activated partial thromboplastin time (APTT) and thrombocytopenia are commonly seen in patients with chronic liver disease, traditionally leading to the perception of increased bleeding risk. Instead, chronic liver disease is associated with reductions in both procoagulant and anticoagulant factors with changes in primary hemostasis, secondary hemostasis, and fibrinolysis resulting in a state of rebalanced hemostasis which is poorly reflected by routine coagulation testing [1]. Consequently, PT, APTT, and platelet count have been demonstrated to be poor predictors of bleeding in the periprocedural setting [1].

Despite this knowledge, patients with cirrhosis are frequently transfused in response to abnormal parameters of hemostasis. A nationwide study of 1313 patients with cirrhosis admitted to hospital found that 30% were transfused with a blood product during admission, of which 39% of transfusions were prophylactic [2]. Given the patient safety and financial implications of blood component administration in patients with cirrhosis, there is increasing focus on rationalizing the use of blood products in this cohort.

Given the poor predictive value of PT/INR, thrombocytopenia and hypofibrinogenaemia, in addition to an awareness that the periprocedural bleeding risk for common procedures is low, updated guidance from the European Association for the Study of the Liver (EASL) and the International Society on Thrombosis and Haemostasis (ISTH) recommend against the use of prophylactic blood products prior to procedures [3,4]. This represents a paradigm shift in comparison to recommendations from earlier guidance published by Society for Interventional Radiology (SIR) in 2012 which recommended INR correction to <1.5 for procedures with moderate or significant bleeding risk, INR <2 if low risk of bleeding and platelet transfusion if platelets <50×10^9^/L for all procedures. Updated SIR guidance in 2019 no longer recommends the use of FFP for correcting INR but suggests vitamin K if the INR is >2.5 prior to high-risk procedures, with platelet transfusion thresholds of 20 × 10^9^/L and 30 × 10^9^/L for low and high-risk procedures respectively, and fibrinogen replacement if fibrinogen <1g/L for any procedures [5]. Of note this guidance, addresses procedures undertaken in interventional radiology, and a significant proportion of procedures in patients with cirrhosis are performed at the bedside and in endoscopy. Periprocedural practice remains variable, driven in part by the poor availability of high-quality randomized control trials and the lack of alternative assessment of hemostasis to predict outcomes.

In this review, we consider low bleeding-risk procedures to be those associated with a <1.5% rate of major bleeding, and high bleeding risk procedures with a >1.5% rate of major bleeding in keeping with current guideline documents [6], [7], [8]. Low bleeding risk procedures account for the majority of invasive procedures in patients with cirrhosis. The PROC-BLeeD international prospective multicentre observational study included hospitalized patients with cirrhosis undergoing nonsurgical procedures. A total of 3,006 nonsurgical procedures were recorded, of which 90.9% were low-risk. In contrast with practice elsewhere, <5% of patients received prophylactic blood components in this study [9]. Prophylactic transfusion is often given in the setting of low-risk procedures. An Australian multidisciplinary survey by Janko et al. evaluated current periprocedure practice for patients with cirrhosis undergoing invasive procedures. This showed variation in the use of prophylactic transfusion as well as varied INR / platelet thresholds for transfusion. About 61% of respondents stated that they would give prophylactic platelet transfusion prior to low-risk procedures if the platelet count were <50 × 10^9^/L and 46% would give fresh frozen plasma (FFP) prior to low-risk procedures if the INR was 2 or above [10]. Given that the majority of procedures are low bleeding risk, the proportion of prophylactic transfusion in this setting is considerable.

Viscoelastic hemostatic assays (VHA) provide a point-of-care test assessing global hemostasis by measuring the properties of whole blood clot formation under low shear conditions. It has therefore been argued that VHAs can help inform personalized transfusion and anticoagulation decisions. Thromboelastography (TEG) now has a suggested role in guiding the provision of blood products during liver transplantation. Indeed two recent systematic reviews demonstrated a reduction in blood product transfusion using a VHA-guided approach compared with transfusion guided by conventional coagulation tests in patients undergoing liver transplantation but with no impact on mortality nor length of ICU or hospital stay [11], [12]. In practice, specific VHA parameters and transplantation algorithms vary depending on centre experience [13]. A recent meta-analysis evaluating 5 randomized controlled trials comparing TEG-guided hemostatic management with standard of care in patients with cirrhosis demonstrated a significant reduction in the use of blood products without an increase in bleeding or mortality [14]. However, this meta-analysis included studies of patients with active bleeding and undergoing liver transplantation. Thus, its conclusions may not be translatable to nonbleeding patients with cirrhosis undergoing invasive procedures.

This review will address the clinical need to critically appraise the limited evidence on the use of VHAs to guide prophylactic treatment in patients with liver disease undergoing procedures and to assess if the impact on management improves clinical outcomes.

### Viscoelastic Hemostatic Assays to Predict Procedure-Related Bleeding

In considering the use of any test to guide clinical intervention, prior validation of the test as a reliable predictor of outcome is of particular importance. We reviewed the evidence examining the role of VHAs in the prediction of hemostasis in our population of interest. We selected studies evaluating the ability of VHA to predict procedure-related bleeding in patients with cirrhosis, including both high and low-bleeding-risk procedures. Three relevant observational studies were identified.

Zanetto et al. provide a recent prospective single-centre cohort study to establish whether TEG5000 can be used to identify patients with cirrhosis at increased risk of procedure-related bleeding [15]. The cohort comprised adults with cirrhosis admitted to hospital with acute decompensated disease. TEG was performed on admission and patients observed for procedure-related bleeding. Bleeding was categorized according to ISTH criteria for non-surgical bleeding [16]. Thirty healthy controls matched for age and sex were recruited, all without a history of acute or chronic disease and not taking antithrombotics, anticoagulants, antibiotics, or hormonal therapy.

A total of 153 invasive procedures were performed in 72 patients. Although not classified by the authors, the listed procedures were predominantly associated with a low bleeding risk (for example, paracentesis n = 94; diagnostic endoscopy/colonoscopy, n = 19) [17]. Seven patients experienced procedure-related bleeding; 4 following large volume paracentesis, of which 2 patients subsequently died, 2 following therapeutic thoracentesis, and 1 following tunneled central venous dialysis catheter insertion. Major bleeding was seen in 3 cases – 2 following paracentesis and 1 following thoracentesis. The median time from recruitment to occurrence of bleeding was 5 days. Patients with cirrhosis compared with controls had significantly longer reaction (R) time, longer coagulation (K) time, smaller α-angle and lower maximum amplitude (MA). In spite of this, the majority of these patients did not bleed. TEG parameters of k-time, α-angle, and MA were also significantly different between the bleeding and non-bleeding groups – all indicative of hypocoagulability in the group with bleeding. The MA particularly discriminated between patients who had major, life-threatening bleeding (all n = 3, MA <30 mm) and those who had mild or no bleeding (all n = 69 and healthy controls n = 30, MA >30 mm). Comorbidities generally thought to be associated with increased bleeding risk such as acute kidney injury (AKI) [18] and bacterial infection [19] were not predictors of bleeding, nor was the severity of cirrhosis. Conventional hemostasis parameters (INR, platelet count) were similar between bleeding and non-bleeding groups, supporting the existing body of evidence that such parameters do not predict bleeding [20].

The authors conclude that TEG parameters associated with hypocoagulability appeared to predict procedure-related bleeding, especially MA <30 mm. The authors also speculate that whilst validation of results with a larger cohort is needed, TEG parameters could potentially identify a threshold to recognize patients with acute decompensation at higher risk for procedure-related bleeding, and hence where preprocedure prophylaxis (fibrinogen and platelets) could be considered.

In comparison to other studies, Zanetto et al examined whether TEG parameters could be used to estimate bleeding risk, without influencing transfusion strategy. A key strength is the inclusion of patients with additional risk factors for bleeding such as infection and AKI which is likely more representative of the cirrhosis population when admitted to hospital. Those admitted with variceal bleeding, or with major bleeding in the 30 days prior to admission, acute on chronic liver failure (ACLF) or admitted to the intensive care unit (ICU) were however excluded. This study also standardized major bleeding with ISTH criteria and recorded occurrence of thrombosis during admission, an often under reported outcome in this population. Patients with a prior history of thrombosis however, were excluded. Given the prevalence of portal vein thrombosis in this population, the cohort studied may not be truly representative of the larger cirrhotic population.

This study records TEG on admission, hence the median time from recruitment to bleeding onset was 5 days. Comparison with a repeat TEG prior to the invasive procedure would have helped ensure stability of TEG parameters over time when assessing their impact on bleeding risk. Another limitation of this study is the use of the TEG®5000 instrument which lacks evaluation of functional fibrinogen.

As this study was observational, patient management was not altered depending on TEG results which were not shared with the clinical team. We are told that a third of patients received pre-procedure blood components, but the criteria for administration are not defined. Amongst the 7 patients with bleeding, 4 received prophylaxis with FFP alone or FFP and platelets. We are not told the breakdown of blood component use in the non-bleeding population. 2 patients died with major bleeding following a low risk procedure (ultrasound guided paracentesis) and both had MA <30 mm and received prophylactic FFP. Whilst MA <30 mm appeared to discriminate between those with major or life-threatening bleeding and those with mild or no bleeding, it remains to be seen whether this indicates an appropriate threshold upon which further management such as transfusion should be guided. As described in this study both patients with life-threatening bleeding and MA <30 mm were given FFP. However, the dose of FFP given was not provided thus any conclusions drawn on the impact of transfusion are limited. This poor outcome despite transfusion raises the question of whether blood component administration in response to abnormal TEG parameters would truly improve outcomes for patients and whether bleeding relates to procedure-related risk factors such as vascular injury, rather than coagulopathy per se. Ultimately as with other studies in this field, the overall low number of bleeding episodes post-procedure is a significant limitation as acknowledged by the authors.

A similar approach is seen by Pandey et al [21], a prospective single-centre observational study to evaluate the validity of TEG to predict postprocedural bleeding after elective central venous cannulation (CVC) in patients with cirrhosis admitted to ICU. Important differences being that all patients underwent a procedure considered "low risk" and none of the patients received prophylactic transfusion. Unlike the study by Zanetto et al where TEG was performed on admission, Pandey et al performed TEG within 6 hours of the procedure.

Ninety patients aged 20 to 70 years old were recruited and Child-Pugh score, TEG, and laboratory parameters recorded within 6 hours prior to the procedure. Groups were divided by the occurrence of postprocedural bleed defined as bleeding requiring additional and unexpected hemostatic measures eg, compression bandage >15 minutes, transfusion requirement, and bleeding requiring an extended hospital stay. Preprocedure parameters were compared to determine their predictive value.

Bleeding was observed in 11/90 (12%) patients with five patients requiring postprocedural transfusion. K time of ≥3.05 min was reported as a significant predictor of bleeding (area under the curve (AUC) 0.694, *P* = .047) and MA of ≥48.8 mm identified as a significant predictor of nonbleeding. Liver disease severity and INR ≥2.6 were also found to be statistically significant predictors of bleeding, although likely not independent of one another as INR is a measure of liver disease severity.

Pandey et al. offer an evaluation of TEG and laboratory parameters as predictors of bleeding risk post low-risk procedure in the absence of any prophylactic transfusion support. In relation to the question of interest of this review, a strength is therefore the primary outcome being the clinically relevant outcome of bleeding, rather than reduction in blood product support, and the ability to explore associations of clinical outcomes with VHA parameters without being confounded by blood product support. The lack of more detailed outcome data such as mortality and length of stay are required to build confidence in the omission of prophylactic transfusion, especially given the bleeding rates seen were higher than expected for a low-risk procedure (<3%) and notably a validated bleeding outcome score was not used. The generalizability of the findings is further limited in part due to the nonultrasound guided CVC approach which is not representative of current practice and, although sepsis and CKD are not exclusions, the baseline characteristics beyond age and gender are not presented for evaluation. CVC insertion using "landmark technique" does arguably present an increased trial of hemostasis which may have highlighted risk factors for bleeding. Nonetheless, Pandey et al. present an alternative approach to characterizing patients at risk of bleeding post low-risk procedure with cirrhosis using clinical, laboratory, and VHA with the omission of prophylactic transfusion. Additionally, such study approaches may contribute to generation and validation of VHA parameters associated with bleeding in patients with cirrhosis.

Pandey et al. consider all patients with an INR >1.5 or platelets <150 × 10^9^/L at increased bleeding risk, referencing Giannini et al [22]. These thresholds are more conservative than current practice and, whilst this does not dictate interventions in this study, it must be considered in interpretation of the poor predictive value of “laboratory coagulopathy”. To overcome this, both laboratory and TEG parameters have been looked at for individual predictive value from which the authors draw their conclusions that TEG was better at predicting bleeding compared to platelet count and INR.

A third study, Rocha et al [23] reported neither TEG nor conventional assays of hemostasis were useful as predictors of ulcer bleeding postendoscopic variceal band ligation (EVL) in 150 patients with cirrhosis, however, an association was found with severity of liver disease (Child-Pugh C). Of note, patients with renal failure and sepsis were excluded from the study.

The findings of these observational studies were varied. Rocha et al did not support a role for TEG in predicting bleeding post-EVL, however, an association between bleeding and severity of liver disease was identified. In contrast Pandey et al identified K time ≥3.05 minutes as a predictor of bleeding and MA ≥48.8 mm as a predictor of nonbleeding post-CVC insertion. Similarly, Zanetto et al found MA <30 mm to be predictive of procedure-related bleeding. These contrasting findings may relate to differences in type of procedure and associated risk factors for bleeding. Further studies to determine the potential predictive value of VHAs are needed using modern cartridge-based technology with validated definitions for bleeding.

### Viscoelastic Devices to Guide Periprocedural Transfusion

Despite limited evidence conferring the ability for VHAs to predict hemostasis and therefore a threshold at which bleeding might be increased, there have been a number of trials translating VHA results to guide hemostatic management periprocedurally. We searched the literature for randomized controlled trials (RCTs) examining the use of VHAs to guide peri-procedural transfusion in patients with cirrhosis. We selected three influential examples of RCTs collectively, including low and high-bleeding risk procedures, first in adults and second in children.

### Adults

De Pietri et al [24] enrolled adults aged 18 to 80 years with cirrhosis scheduled for an invasive procedure. Patients with ongoing bleeding, previous or current thrombotic events, antiplatelet/anticoagulant therapy, infection/sepsis, or hemodialysis in the preceding 7 days were excluded. Sixty patients were randomized equally to TEG 5000 guided transfusion or standard of care (SOC). Both low and high-risk procedures were included with an approximately equal split of each in both TEG and SOC groups. Blood products were administered as per thresholds in Table 1 (FFP according to R time and INR, platelets for MA, or platelet count). The primary endpoint was blood product use. Secondary end points included bleeding complications (as per WHO bleeding score), transfusion-related side effects, other procedure-related complications. Following discharge, weekly follow-up assessment calls were made for 90 days postprocedure.

**Table 1:** Comparison of hemostasis indices used by included studies

Author, year	Population	Procedure	Thresholds for transfusion in randomized controlled trials											
			Laboratory parameters in SOC arm				TEG®5000			ROTEM				
			INR*	APTT* (s)	Platelets† (x10^9^/L)	Fibrinogen‡ (mg/dL)	R* (min)	MA† (mm)	K time (min)	CT_EXTEM_* (s)	A10_EXTEM_§≈ (mm)	A10_FIBTEM_§≈ (mm)	MCF_EXTEM_† (mm)	MCF_FIBTEM_‡ (mm)
Randomized controlled trials:														
De Pietri et al, 2016 [24]	Adults with cirrhosis	Low & high risk	>1.8	N/A	<50	N/A	>40	<30	N/A	N/A	N/A	N/A	N/A	N/A
Rocha et al, 2020 [25]	Adults with cirrhosis in ICU	CVC (low risk)	>1.5 (>5)^a^	>50	<50 (<25)^a^	<150	N/A	N/A	N/A	>80	EXTEM <40 and FIBTEM ≥10§ EXTEM <40 and FIBTEM <10≈		N/A	N/A
Maria et al, 2022 [26]	Children <18yrs with cirrhosis	Low & high risk	>1.5	N/A	<50 (<60 liver biopsy)	<80	N/A	N/A	N/A	>80	N/A	N/A	<35	<7
Observational studies:			Proposed predictors of bleeding^b^/non-bleeding^c^											
Pandey et al, 2017 [21]	Adults with cirrhosis in ICU	CVC (low risk)	b>2.6	N/A	N/A	N/A	N/A	c>48.8	b>3.05	N/A	N/A	N/A	N/A	N/A
Zanetto et al, 2021 [9]	Adults with cirrhosis in hospital ward	Low & high risk	N/A	N/A	b<50 not predictive	N/A	N/A	b<30	N/A	N/A	N/A	N/A	N/A	N/A

A10, Amplitude at 10 minutes; APTT, activated partial thromboplastin time; CT, Clotting time; CVC, central venous catheter; extem, extrinsic pathway thromboelastography; fibtem, fibrinogen thromboelastography; ICU, intensive care unit; INR, International Ratio; MA, maximum amplitude; MCF, maximum clot firmness; N/A, not assessed; ROTEM, rotational thromboelastography; SOC, standard of care; TEG, thromboelastometry.

⁎ Thresholds for FFP transfusion in RCTs.

† Thresholds for platelet transfusion in RCTs.

‡ Thresholds for cryoprecipitate.

§ Platelet transfusion threshold if A10extem <40 and A10fibtem ≥10.

≈ Cryoprecipitate threshold if A10extem <40 and A10fibtem <10.

a Thresholds in restrictive transfusion arm.

b Proposed predictor of bleeding in observational studies.

c Proposed predictor of non-bleeding in observational studies.

All patients in the SOC group were transfused; 53.3% FFP, 33.3% platelets, 13.3% both. In comparison, 5 patients in TEG group were transfused (16.7%) – 2 received platelets, 3 FFP and platelets. The criteria for transfusion were the same regardless of procedure risk and the total amount of FFP and platelets transfused based on TEG was higher with low-risk procedures vs high-risk procedures.

Post-procedure bleeding was seen in 1 patient in SOC group following large-volume paracentesis. Blood parameters were repeated postprocedure and showed a significant drop in Hb and INR in the SOC group. The drop in Hb may represent hemodilution in the context of FFP administration as clinical evidence of bleeding was seen in 1 patient only. One allergic reaction to FFP was recorded in the SOC group, whilst none were observed in the TEG group.

This study demonstrates lower transfusion rates when guided by TEG without a consequent increase in bleeding. The authors suggest that even in patients with significant coagulopathy, procedure-related bleeding is rare, therefore TEG thresholds should be re-evaluated.

Although the rate of transfusion was lower in the high risk procedure group, the risk of procedure-related bleeding did not increase. The authors highlight that the very low rate of bleeding seen, and the fact that the patient who bled had a low risk procedure with FFP preprocedure suggests that bleeding is not related to coagulopathy itself but rather local complications of the procedure eg, damage to abdominal wall blood vessels. Similarly, Zanetto et al. found life-threatening bleeding in 2 patients with low risk procedures despite prophylactic FFP.

The 100% preprocedure transfusion rate in the SOC group was in keeping with published Society of Interventional Radiology guidance at the time, but out of keeping with contemporary guidance recommendations [4], [5], [6], [7]. When considering the TEG-guided transfusion group, whilst a significant reduction in the rate of blood component use was seen, one could question whether more restrictive TEG parameters resulting in even lower rates of transfusion could be appropriate given the overall low incidence of procedure-related bleeding. Notably patients with infection/sepsis were excluded from this trial. As mentioned previously, given the frequent occurrence of infection in patients with chronic liver disease, particularly those undergoing paracentesis for exclusion of spontaneous bacterial peritonitis, it would be beneficial to establish whether similar results are seen in this cohort. A further limitation of this trial is that it was grossly underpowered to reach conclusions regarding bleeding complications.

Addressing the limitations of the SOC comparator arm as being non-representative of current practice, Rocha et al. 2020 conducted a RCT comparing 3 different transfusion protocols in patients with cirrhosis prior to CVC placement, a low-risk procedure [25]. Fifty-seven adults >18 years with liver cirrhosis admitted to the ICU at a single urban tertiary medical center were enrolled. Patients with acute liver failure, Von Willebrand Disease, and those on treatment dose anticoagulation were excluded. CVC placement was under US guidance by a trained operator and the location of CVC remained at the operator's discretion (jugular, femoral, subclavian).

The transfusion protocol for the standard of care arm used 10 mL/kg of FFP if INR >1.5 or APTT >50 seconds, 1 unit per 10 kg of random platelets (up to 10 units) or one apheresis platelets if platelets <50 × 10^9^/L and/or one unit per 10 kg of cryoprecipitate (up to 10 units) if fibrinogen <150 mg/dL. In the restrictive transfusion protocol, 10 mL/kg of FFP was given if INR >5, and/or 1 unit per 10 kg of random platelets (up to 10 units) or one apheresis platelet if platelet count <25 × 10^9^/L. The TEG-guided protocol used 10 mL/kg FFP if CT_EXTEM_ >80 seconds, and/or 1 unit per 10 kg of random platelets (up to 10 units) or one apheresis platelets if A10_EXTEM_ <40mm and A10_FIBTEM_ ≥ 10mm, or 1 unit per 10 kg of cryoprecipitate (up to 10 units) if A10_EXTEM_ <40 mm and A10_FIBTEM_ <10mm. These protocols are summarized in Table 1.

The primary endpoint was the proportion of patients transfused with any blood component and secondary endpoints included incidence of bleeding and transfusion-related adverse events. Major bleeding was defined as overt bleeding with any of a decrease of 2 g/dL or more in hemoglobin; transfusion of 2 or more units of packed red blood cells with no increase in hemoglobin level; a decrease in systolic blood pressure by 10 mm Hg or more while patient is sitting up; a spontaneous decrease in systolic blood pressure of 20 mm Hg or more; or an increase in heart rate by 20 beats per minute or more; bleeding at any one of the following sites: pleural, pericardial or retroperitoneal; or wound-related bleeding requiring medical intervention.

Median platelet count for all groups was >40 × 10^9^/L. The results showed that there were no cases of major bleeding in any of the patients and 28-day mortality was similar across all groups. A restrictive transfusion strategy could reduce blood product use and costs associated with critically ill cirrhosis patients undergoing CVC, with only 3 (15.8%) patients receiving blood product support, however, a VHA-based strategy was no different from usual care with regards to use of blood products (13 (68.4%) vs 14 (73.7%) respectively). A summary of blood component administration and procedure-related bleeding rates in relevant RCTs is shown in Table 2.

**Table 2:** Comparison of bleeding and transfusion outcomes in randomized controlled trials.

RCT	Patients receiving any component transfusion^a^			Procedure-related major bleeding^a^			VHA device used
	VHA arm	SOC arm	Restrictive transfusion arm	VHA arm	SOC arm	Restrictive transfusion arm	
Maria et al, 2022 [26]^b^	14/30 (46.7%)	30/30 (100%)	N/A	1/30^b^ (3.3%)	2/30^b^ (6.7%)	N/A	ROTEM
De Pietri et al, 2016 [24]	5/20 (16.7%)	30/30 (100%)	N/A	0/30 (0%)	1/30 (3.3%)	N/A	TEG®5000
Rocha et al, 2020 [25]	13/19 (68.4%)	14/19 (73.7%)	3/19 (15.8%)	0/19 (0%)	0/19 (0%)	0/19 (0%)	ROTEM

N/A, not applicable; RCT, randomized controlled trial; SOC, standard of care; VHA, viscoelastic hemostatic assay.

a Variables expressed as number and percentage.

b Figures represent all bleeds as major bleeding not defined by authors.

This is a well-designed study where the inclusion of a restrictive transfusion group helps to demonstrate the low overall risk of postprocedure bleeding in the setting of US guided CVC placement in critically ill cirrhosis patients. This also highlights the argument that whilst numerous studies present findings that VHAs can reduce blood product use compared to conventional assays of hemostasis, VHAs may still not identify a population who benefit clinically from blood product support, but rather support the rationale that less or even omission of transfusion may be non-inferior to transfusion. The study is however underpowered due to transfer of the liver transplantation programme to another hospital, resulting in slow recruitment and early termination, and must be considered with regards to conclusions drawn of bleeding and other clinically relevant secondary outcomes, as the authors note. This, in combination with the conservative rotational thromboelastometry (ROTEM) parameters used to guide transfusion, may help to explain why no difference was found between thromboelastometry and SOC groups. A strength of the study is that patients with sepsis and chronic kidney disease, recognized as independent risk factors for bleeding and common amongst the cirrhotic population, are included and balanced between groups. Although no sensitivity or multivariable logistic regression analysis is performed, bleeding rates remain low overall despite inclusion of these patients, supporting their incorporation in future trials and allowing findings to be more generalizable to the populations treated.

### Children

Maria et al [26] conducted a single-centre randomized controlled trial with aim of evaluating efficacy and safety of ROTEM-based transfusion in children with cirrhosis having invasive procedures. Children <18 years with cirrhosis, INR 1.5 to 2.5 and platelet count 20 to 50 × 10^9^/L were included. In patients undergoing liver biopsy a platelet count of 40 to 60 × 10^9^/L was required. Sixty patients were included, with equal numbers in the ROTEM and “conventional” care groups. Baseline characteristics were similar in the two groups and there was no loss to follow up.

According to the protocol, patients would receive FFP 15 mL/kg, platelets 10 mL/kg, and cryoprecipitate 5 mL/kg if thresholds considered as increased bleeding risk were met (Table 1). Minimum follow up period postprocedure on the ward was 24 hours.

Patients undergoing both low and high-risk procedures were included. Those classified as low risk included central venous cannulation, hemodialysis catheter insertion, ascitic or pleural tap, and endoscopic variceal ligation. About 19 (62.3%) in the ROTEM group and 20 (66.7%) in the conventional group underwent procedures with a low bleeding risk. The primary outcome measure was volume of total blood components transfused. Secondary outcome measures were comparison of proportion of patients requiring blood components, volume of each component transfused, procedure-related bleed, rate of transfusion reactions and cost-effectiveness.

Median total volume of transfused blood components was significantly lower in the ROTEM group compared with the conventional management group (*P* < .001). The number of children receiving any blood component was 46.7% in the ROTEM group compared with 100% in the conventional group (*P* < .001) as summarized in Table 2. When considering FFP transfusion specifically, 43.3% in the ROTEM group were transfused compared with 83.3% in the conventional arm (*P* = .001). Platelet transfusion rate was also significantly lower in the ROTEM group compared with the conventional group (6.7% vs 40%, *P* = .002). Cryoprecipitate transfusions were not significantly different between the two groups.

The rate of procedure-related bleeding (summarized in Table 2) was not significantly different between the two arms, although absolute numbers were small (1 bleed in ROTEM group, 2 in conventional group). Notably the bleed in the ROTEM group was with abdominal drain insertion, classified as a high risk procedure in this paediatric setting. Two bleeds in the conventional care group were with therapeutic abdominal paracentesis, considered low risk. The authors do not define criteria for minor versus major bleeding, nor the definition of procedure-related bleeding. Transfusion complications were not seen in either group. The authors conclude that the use of ROTEM prior to invasive procedures was cost-effective and resulted in lower rates of blood transfusion without associated increase in procedure-related bleeding.

This trial is the first of its kind in the paediatric population, adding evidence to this often under studied population. A strength of this study is the inclusion of patients with acute kidney injury or sepsis, as these comorbidities are associated with a predisposition to bleeding in the context of cirrhosis. Furthermore, there was an equal representation of low and high risk procedures in both arms of the trial.

Whilst the reduction in rates of blood component transfusion without consequent increase in bleeding in the ROTEM guided group is reassuring, an important limitation of this study is whether the “conventional” arm is an appropriate reflection of standard of care. One could argue that 100% rate of blood component transfusion in the conventional arm, even for low risk procedures signifies a liberal approach to transfusion which may not be appropriate and is not in line with multiple societal recommendations for adults, including ISTH [4]. The authors do not subdivide their results based on bleeding risk for the procedure so it is not possible to comment on the transfusion rate in the ROTEM arm for low risk procedures specifically. Furthermore, this study is underpowered to reach conclusions regarding clinically relevant outcomes such as bleeding, limiting the strength of the conclusions drawn.

Although lower than the conventional group, 46.7% of patients in the ROTEM group still received transfusion. Given the overall low rate of bleeding complications one could question whether similarly low bleeding rates would be seen with more stringent ROTEM-based criteria resulting in an even lower rate of transfusion overall. The authors report that if ROTEM or conventional parameters were abnormal resulting in transfusion that the relevant tests were repeated following the procedure, however data on changes in these parameters following transfusion are not provided. It would be interesting to assess what impact transfusion has on these parameters.

De Pietri et al and Maria et al demonstrate a reduction in the use of blood components peri-procedurally when guided by VHA parameters rather than standard of care protocols in place at that time, without a consequent increase in bleeding. Rocha et al went further in comparing a restrictive transfusion strategy alongside standard of care and TEG-guided transfusion. Transfusion rates were significantly reduced with the restrictive transfusion strategy only, again without major bleeding in any group.

Whilst these studies suggest VHA use can reduce blood components transfused, the standard of care arms used for comparison are now outdated. The lack of validated thresholds for transfusion, and inadequate power to evaluate any impact on procedural bleeding remain major limitations.

## Discussion

The studies reviewed collectively demonstrate the rationale and potential for VHAs to guide periprocedural hemostasis management in cirrhosis, whilst highlighting significant limitations in existing evidence to include a lack of validated VHA parameters and need for comparisons with up-to-date transfusion practices. Further studies, in the form of large observational studies followed by adequately powered RCTs, are required to detect impact on clinical outcomes which are of relevance to patients, such as bleeding, transfusion reactions, and mortality, rather than solely frequency or volume of transfusion. We acknowledge delivery of RCTs with appropriate clinical endpoints is a significant challenge given the low event rate of clinically relevant outcomes, particularly following procedures associated with a low bleeding risk. Future studies should therefore focus on procedures associated with a high bleeding risk.

Given the significant limitations of traditional coagulation parameters in reflecting rebalanced hemostasis, VHAs present a potentially appealing alternative with the ability to capture the dynamic properties of whole blood clot formation whilst delivering real-time results. In line with this, studies have demonstrated TEG parameters to be generally preserved in the majority of patients with cirrhosis [27]. There are however, important limitations to consider. VHAs lack activation of the protein C pathway. Protein C activity is typically reduced in cirrhosis which can result in hypercoagulability which is not reflected by VHAs. VHAs are also insensitive to altered von Willebrand factor levels, which are typically raised in patients with cirrhosis and felt to be compensatory for the effects of thrombocytopenia. In combination this suggests that VHAs may underestimate the hemostatic potential in patients with cirrhosis [28]. One could therefore argue that a transfusion strategy based on VHAs may result in unnecessary use of blood products. As such, there remains high variability of what is defined as “coagulopathy” by VHA indices used in the literature (Table 1) and disagreement between studies as to the predictive reliability. To derive standardized and validated VHA parameters associated with bleeding, studies should be transparent in the rationale by which they determine values used and signpost the reader and other researchers to the supporting evidence. In the studies presented here, Zanetto et al. identify TEG5000 MA <30mm as predictive of bleeding which is the threshold utilized by De Pietri et al. in their earlier work, and Rocha et al. make a point to reference the origin of the ROTEM parameters applied [29]. Pre- and posttransfusion VHA changes could also help shed light on the relevance of such parameters and the impact of transfusion. Finally, consideration must be given to the methodology of the assays used. The required pipetting of reagents and blood with older technology gives an opportunity for significant inter- and intraoperator variability and studies utilizing newer cartridge-based methods eg, TEG6S are necessary to identify if findings will differ [30]. The use of TEG6s and TEM in future studies also has the advantage of a functional fibrinogen measurement compared with older methods such as TEG5000. None of the studies discussed specifically considered fibrinogen replacement. There is very limited data evaluating fibrinogen as a predictor of bleeding in patients with cirrhosis. The 2012 Society of Radiology guidance recommended a threshold of fibrinogen of <1 g/dL which would correlate with a FIBTEM of 5 to 7 mm. Maria et al utilized this threshold, de Pietri did not use fibrinogen replacement and Rocha et al used a higher threshold (fibrinogen <1.5 g/dL) for SOC and a combined threshold of A10_EXTEM_ < 40 mm and A10_FIBTEM_ < 10 mm for fibrinogen replacement (see Table 2).

A VHA guided strategy led to a significant reduction in the rate of transfusion with any component in two of the RCTs reviewed (Table 2) [24,26]. Whilst a lack of increased bleeding events despite a reduction in rates of transfusion as demonstrated in Table 2 may be viewed as positive, the key question is whether the comparator SOC arms (in keeping with published guidance at the time of study) remain relevant to current practice, particularly given the recent PROC-BLeeD findings in which <5% of 3,006 procedures performed in hospitalized patients received plasma or platelet transfusion support [5,31]. Table 1 highlights the variation in conventional parameters in the SOC arms, all of which would be out of keeping with recommendations in contemporary, international periprocedural management guidelines [3,4].

The inclusion of a restrictive transfusion arm in the study by Rocha et al [25] resulted in reduced rates of transfusion when compared with a VHA-guided approach, again without any increase in major bleeding. This suggests that a VHA-guided approach, particularly in the setting of low-risk procedures may expose patients to unnecessary transfusion and consequent risks without improving clinical outcomes.

Specific to the low risk procedure setting, low rates of peri-procedural major bleeding seen overall (Table 2), combined with the lack of studies limited to low risk procedures only, make it difficult to draw firm conclusions. As highlighted previously, future studies should examine the use of VHAs in the context of restrictive transfusion practices in keeping with contemporary guidance and present data stratified by procedure risk. Whilst the definition of cirrhosis is consistent between studies, patients with other important risk factors for bleeding, such as infection and renal impairment, are often underrepresented or excluded. Studies should take care to maintain generalisability of findings to the population of interest.

A summary of upcoming and currently recruiting trials examining the role of VHAs in the periprocedural setting in cirrhosis is presented in Table 3. The majority of studies focus on the amount of blood components transfused as their primary outcome, rather than clinically relevant outcomes such as the incidence of bleeding, with the exception of Janko et al [32]. However, given the target recruitment of 56 participants or procedures in this study and the anticipated low incidence of bleeding, this will not be adequately powered to address this. Aside from one study (NCT05672589), the majority of trials have a low target recruitment size, thus likely making them underpowered to answer clinically relevant questions regarding bleeding or transfusion complications, given their low incidence. Two of the studies listed in Table 3 (NCT05698134, ACTRN12619000644167) are also limited by a lack of standardization in the standard of care arm, instead allowing INR and platelet thresholds for transfusion to be determined by usual practice at participating sites [32]. As noted by the European Association for the Study of Liver disease, studies to determine the role of VHAs in predicting procedural bleeding are required.

**Table 3:** Current research studies in progress evaluating the role of thromboelastometry/thromboelastography in patients with cirrhosis undergoing procedures.

Study title & clinical trials identifier	Study status	Size of study	Location	Population	Thresholds for intervention	Outcomes
To compare relaxed rotational thromboelastometry cut-offs with standard cut-offs for guiding blood product use before invasive procedures in cirrhosis and acute on chronic liver failure patients (NCT05672589)	Recruiting	1,050 participants	India	Aged >18 y with cirrhosis and/or ACLF scheduled to undergo a high risk procedure	Relaxed ROTEM criteria: EXTEM CT >90s, FFP/PCC* EXTEM MCF<30 mm and FIBTEM MCF <7 mm, cryoprecipitate* EXTEM MCF <30 mm and MCF FIBTEM ≥7 mm, platelets* Standard ROTEM criteria: EXTEM CT >80s, FFP/PCC* EXTEM MCF<35 mm and MCF FIBTEM <8 mm, cryoprecipitate* EXTEM MCF <35 mm and FIBTEM MCF ≥8 mm, platelets*	Primary: To compare the proportion of patients requiring any blood products transfusion Secondary: To compare the proportion of patients requiring FFP / platelets / cryoprecipitate / tranexamic acid infusion To compare amount of FFP / platelets / cryoprecipitate transfused To compare occurrence of bleeding To compare transfusion-related side effects To compare cost incurred
ROTEM guided transfusion for elective procedures in patients with cirrhosis (REduCe): An open label randomized controlled trial (NCT05698134)	Recruiting	74 participants	Singapore	Aged >21 y with cirrhosis and acute decompensation / ACLF / acute liver failure undergoing selected elective low and high-risk procedures	TEM guided group: Parameters for transfusion not specified Standard of care group: Transfusion of blood products based on prevailing institution protocol, based on platelet count, APTT and PT/INR	Primary: Difference in amount of blood products transfused Secondary: Periprocedural bleeding complications Transfusion-related adverse events Hospital length of stay 30-d and 90-d survival Thrombotic complications Procedure-related complications other than bleeding
Peri-interventional coagulation management of patients undergoing a TIPS (NCT04421924)	Recruiting	39 participants	Austria	Aged >18 y with liver cirrhosis with an indication for TIPS	TEG group: R-time >40 min, PCC 10IE/kg MA <30 mm, 1 unit apheresed platelets Standard of care group: PT <50% and/or INR >1.8, PCC 10IE/kg Platelets <50 × 10^9^/L, 1 unit apheresed platelets	Primary: Amount of blood products (coagulation factors and platelets) transfused for preinterventional correction of coagulation status Secondary: Bleeding, complications, mortality, modified TIPS score (all within 90 d) Factor XIII activity levels (within 2 d) compared with TEG parameters
Rotational thromboelastometry-guided blood product in patients with cirrhosis undergoing invasive procedures (ACTRN12619000644167)[32]	Recruiting	56 participants or procedures	Australia	≥ 18 y with cirrhosis, planned for a low or high bleeding risk invasive procedure	TEM group: EXTEM A_5_ <35 mm & FIBTEM A_5_ <9 mm: Give 1 dose cryoprecipitate EXTEM A_5_ <35 mm & FIBTEM A_5_ >9 mm Give 1-2 bags platelets EXTEM CT >80 s, transfuse 1 unit FFP if cryoprecipitate also indicated OR 10-15 mL/kg FFP if no cryoprecipitate indicated Standard of care group: FFP/ platelets/ cryoprecipitate for INR and platelet thresholds at discretion of individual participating sites	Primary: Proportion of procedures requiring prophylactic transfusion Procedure-related bleeding complications Secondary: Amount of FFP/platelets/cryoprecipitate transfused Transfusion-related side effects Procedure-related complications other than bleeding Hospital length of stay and survival
Rotational thromboelastometry versus conventional hemostatic tests in children with decompensated cirrhosis undergoing invasive procedures (NCT05734001)	Not yet recruiting	90 participants	India	Children aged 6 mo – 18 y with decompensated cirrhosis undergoing low or high risk procedures	TEM group: EXTEM CT >80 s, 15 mL/kg FFP MCF <35 mm, 10 mL/kg platelets FIBTEM MCF <7 mm, 5 mL/kg cryoprecipitate Standard of care group: INR >2.5, 15 mL/kg FFP Platelet count 20-50 × 10^9^/L, 10 mL/kg platelets Fibrinogen <80 mg/dL, 5 mL/kg cryoprecipitate	Primary: Amount of total component transfused Secondary: Amount of FFP / platelets / cryoprecipitate Bleeding rate Rate of transfusion reactions

A_5_, amplitude at 5 minutes; ACLF, acute on chronic liver failure; APTT, activated partial thromboplastin time; CT, clotting time; FFP, fresh frozen plasma; INR, international normalized ratio; MA, maximum amplitude; MCF, maximum clot firmness; PCC, prothrombin complex concentrate; PT, prothrombin time; TEG, thromboelastography; TEM, thromboelastometry; TIPS, transjugular intrahepatic portosystemic shunt.

⁎ proposed dose not published.

## Conclusions

There is limited evidence to support VHA as predictive of procedural bleeding in patients with chronic liver disease. Therefore, the reduction in periprocedural blood component support associated with VHA use cannot be assumed to improve hemostasis or clinical outcomes. Procedure-related major bleeding rates were low both in the setting of more liberal and restrictive transfusion strategies presented in the studies discussed. Moreover, the recent PROC-BLeeD study of >1,000 hospitalized patients undergoing procedures confirmed a low major bleeding rate of <1%, and that conventional laboratory parameters did not predict bleeding (with <5% receiving plasma or platelets), supporting contemporary guidance to not evaluate or correct conventional hemostatic parameters. We suggest a large prospective collaborative observational study, in patients with cirrhosis undergoing *high risk* procedures, is required to derive and validate VHA parameters associated with an increased risk of procedural bleeding in patients with cirrhosis. Should such parameters be identified, appropriately powered interventional studies using VHA-guided transfusion compared with a restrictive strategy could then be justified, noting that such a study will be challenged by a large sample size given the low of incidence of procedural bleeding (anticipated procedural major bleeding ∼5% following a high risk procedure) [31].

The current literature review supports the restrictive transfusion practice advocated in guidance documents recommending prophylactic transfusion in patients with cirrhosis undergoing procedures.

## Funding

This work is supported by the NIHR Blood and Transplant Research Unit in Data Driven Transfusion Practice (NIHR203334) and 10.13039/501100000265Medical Research Council (MR/W030292/1). The views expressed are those of the author(s) and not necessarily those of the NIHR or the Department of Health and Social Care. These funding sources had no involvement in design of this article, writing of the report or the decision to submit the article for publication.

## Author Contributions

All authors were involved in conceptualization. SM and EM wrote the original draft, LR and SS reviewed and edited original manuscript. All authors approved the final manuscript for submission.

## Declaration of competing interest

The authors declare no conflicts of interest.
